# Etiology of Acute Febrile Illness in the Peruvian Amazon as determined by modular formatted quantitative PCR: A Protocol for RIVERA, a Health Facility-Based Case-Control Study

**DOI:** 10.21203/rs.3.rs-2635774/v1

**Published:** 2023-03-31

**Authors:** Pablo Peñataro_Yori, Maribel Paredes_Olórtegui, Francesca Schiaffino, Karin Perez, Greisi Curico_Huansi, Thomas Flynn, Jixian Zhang, Cesar Ramal_Asayag, Graciela Meza_Sanchez, Hermann Silva_Delgado, Martin Casapia_Morales, Wilma Casanova, Bruce Jiu, Cesar Munayco_Escate, Rachel Silver, Olga Henao, Kerry K. Cooper, Jie Liu, Eric Houpt, Margaret N Kosek, Josh M Colston, Richard Oberhelman, Tackeshy Pinedo_Vasquez, Paul F Garcia_Bardales, Wagner Valentino Shapiama_Lopez, Loyda Fiorella Zegarra_Paredes

**Affiliations:** University of Virginia; Asociación Benfica Prisma; Universidad Peruana Cayetano Heredia; Asociación Benfica Prisma; Asociación Benfica Prisma; University of Virginia; University of Virginia; Universidad Nacional de La Amazonia Peruana; Universidad Nacional de La Amazonia Peruana; Universidad Nacional de La Amazonia Peruana; Universidad Nacional de La Amazonia Peruana; Universidad Nacional de La Amazonia Peruana; Direccion Regional de Salud, Loreto; Centro de Epidemiologia, Prevencion, y Control de Enfermedades, Ministerio de Salud; Centers for Disease Control and Prevention; Centers for Disease Control and Prevention; University of Arizona; Qingdao University; University of Virginia; University of Virginia; University of Virginia; Tulane School of Public Health; Asociación Benfica Prisma; Asociación Benfica Prisma; Asociación Benfica Prisma; Asociación Benfica Prisma

**Keywords:** Loreto, acute febrile illness, whole blood, TaqMan, active surveillance, case-control, population attributable fraction

## Abstract

**Background:**

The study of the etiology of acute febrile illness (AFI) has historically been designed as a prevalence of pathogens detected from a case series. This strategy has an inherent unrealistic assumption that all pathogen detection allows for causal attribution, despite known asymptomatic carriage of the principal causes of acute febrile illness in most low- and middle-income countries (LMICs). We designed a semi-quantitative PCR in a modular format to detect bloodborne agents of acute febrile illness that encompassed common etiologies of AFI in the region, etiologies of recent epidemics, etiologies that require an immediate public health response and additional pathogens of unknown endemicity. We then designed a study that would delineate background levels of transmission in the community in the absence of symptoms to provide corrected estimates of attribution for the principal determinants of AFI.

**Methods:**

A case-control study of acute febrile illness in patients ten years or older seeking health care in Iquitos, Loreto, Peru, was planned. Upon enrollment, we will obtain blood, saliva, and mid-turbinate nasal swabs at enrollment with a follow-up visit on day 21–28 following enrollment to attain vital status and convalescent saliva and blood samples, as well as a questionnaire including clinical, socio-demographic, occupational, travel, and animal contact information for each participant. Whole blood samples are to be simultaneously tested for 32 pathogens using TaqMan array cards. Mid-turbinate samples will be tested for SARS-CoV-2, Influenza A and Influenza B. Conditional logistic regression models will be fitted treating case/control status as the outcome and with pathogen-specific sample positivity as predictors to attain estimates of attributable pathogen fractions for AFI.

**Discussion:**

The modular PCR platforms will allow for reporting of all primary results of respiratory samples within 72 hours and blood samples within one week, allowing for results to influence local medical practice and enable timely public health responses. The inclusion of controls will allow for a more accurate estimate of the importance of specific, prevalent pathogens as a cause of acute illness.

**Study Registration:**

Project 1791, Registro de Proyectos de Investigación en Salud Pública (PRISA), Instituto Nacional de Salud, Perú.

## Introduction

Numerous febrile illnesses and emerging infections are endemic to the tropical regions of South America and are among the etiologies underlying the clinical syndrome of Acute Febrile Illness (AFI) [1]. For example, in the Peruvian Amazon, Plasmodium spp [2–8], dengue virus [9–11], Zika virus, Chikungunya virus, Bartonella sp [12], Leptospira [13–16], Rickettsiae [17–19], Oropouche virus [20] and Mayaro virus [21, 22] co-exist alongside other pathogens and are impossible to differentiate on clinical presentation. While differential diagnostic methods for several etiologies of AFI have improved in recent years - rapid tests for malaria and dengue being notable examples - they remain inadequate for most pathogens in resource-constrained settings [23, 24]. Furthermore, for some agents such as Rickettsia, Anaplasma and Ehrlichia, no real-time well-standardized diagnostics are available at the regional level [17, 18, 25–27].

In recent years, TaqMan Array Cards (TACs) - modular semi-quantitative polymerase chain reaction (PCR) diagnostic devices - have demonstrated the capacity to enhance disease surveillance through their efficiency and ease of use [28, 29]. However, this technology has only been limited to AFIs as part of a sustained febrile surveillance program [30].

While blood is generally sterile, microbial nucleic acid can be detected in a certain fraction of healthy individuals even with aseptic collection techniques. Therefore, as molecular diagnostics are applied to AFI patients, the possibility of subclinical detections in immune hosts must be evaluated. Rates of subclinical detection may vary by pathogens, such that some pathogens are never found in asymptomatic individuals and are always considered the attributable etiologic agent when detected. In contrast, detections of other pathogens need to be reconsidered carefully and compared to detections among controls, such as *Plasmodium,* which has a community prevalence of 2–5% in communities around Iquitos [31, 32]. Asymptomatic prevalence of dengue virus (DENV) can reach 5–7.5% in endemic populations [33], while for Zika virus (ZIKV), rates of 60% have been observed [34], and asymptomatic human infection with Leptospira has also been documented [13]. Additionally, estimating the prevalence and microbial load in cases of asymptomatic carriage is essential in understanding the transmission dynamics of the high-burden AFI etiologies.

Given the lack of combined rigorous case definitions and poor understanding of the relative performance of accepted AFI gold standards against emerging and promising diagnostics, this study, named RIVERA, aims to fill a critical knowledge gap to attain more accurate regional and estimates for AFI in the Peruvian Amazon.

## Methods

### Study Aim:

The aim of this study will be to implement year-round, active, etiology-specific surveillance of AFI cases and their age-matched controls in Loreto, Peru, using TaqMan array cards for the detection of bloodborne pathogens. The evaluation of respiratory testing by RT-PCR of Influenza and SARS-CoV-2 was added, given the heavy burden of SARS-CoV-2 in the region [35].

### Study Design:

This study has a prospective, health facility-based, case-control design with active surveillance of AFI in patients (cases) aged ten years or older seeking care at selected facilities in Iquitos, Loreto, Peru, and a nearby rural community. The study will recruit 1,200 cases and controls per year over a four-year period.

### Study Setting:

**Figure 1:**
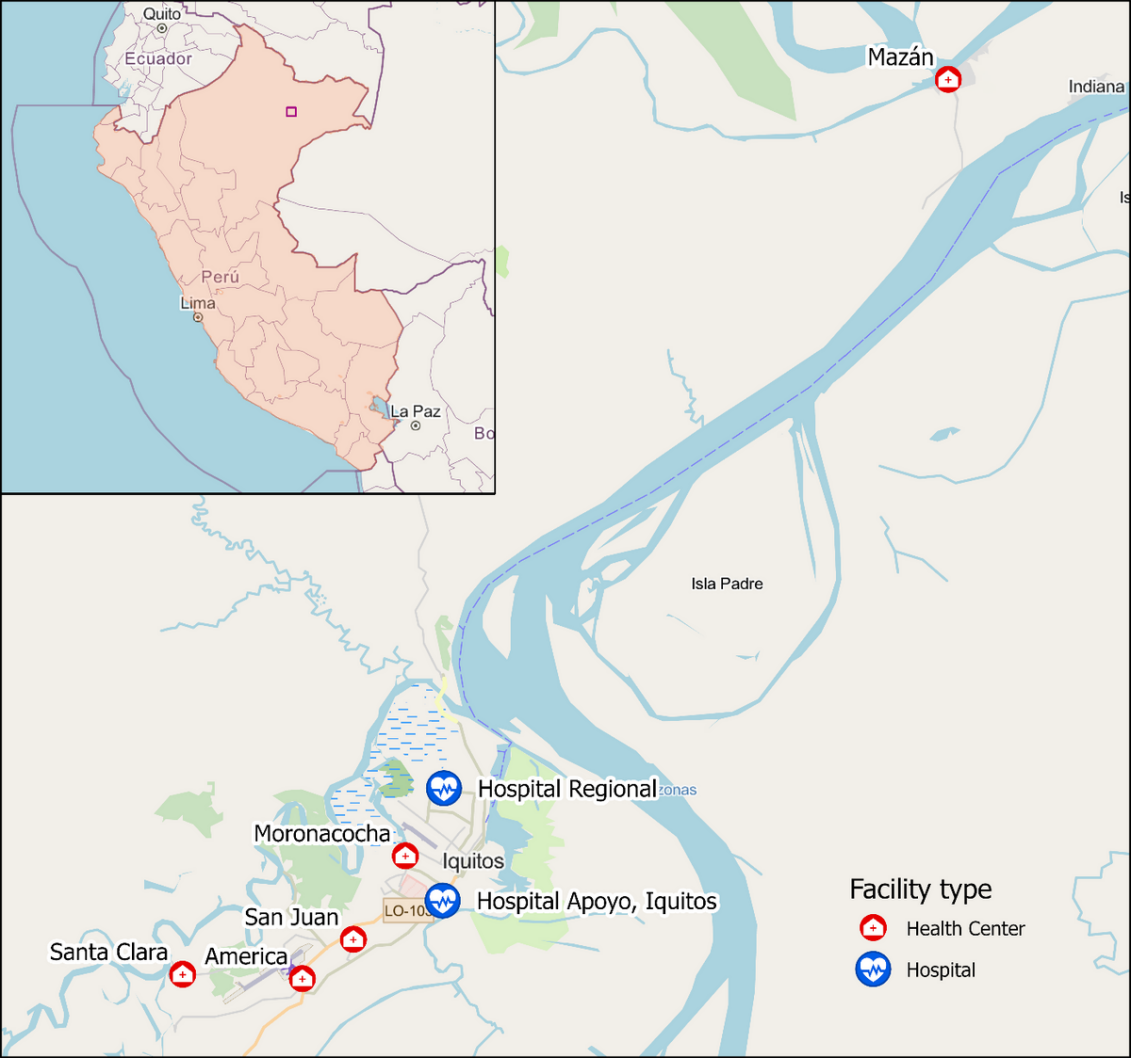
Locations of recruiting health facilities in the Greater Iquitos Metropolitan Area

This study will be carried out at seven health centers distributed in the city of Iquitos, in the province of Maynas, Loreto Region, Peru. These include two tertiary level hospitals (Hospital Regional de Loreto and Hospital Apoyo Iquitos) and four primary care centers (Centros de Salud - C.S.) - C.S. San Juan, C.S. Moronacocha, C.S. Americas, and C.S. Santa Clara - in Iquitos, with an additional fifth health center located some distance downriver in the riverine community of Mazan (C.S. Mazan). Figure 1 shows the locations of the seven recruitment centers. Socioeconomic and health indicators of the region of Loreto lag behind those of the rest of the country, including access to water and sanitation, per capita income, and critical health indicators, including infant mortality (29.5 vs. 12.0 per 1000 in Lima)[36] and rates of childhood stunting of 25.2 (national average 12.1%). In 2021 Loreto reported 85.70% of the total number of cases of malaria in Peru (18,074), and 11.42% of all reported cases of dengue (44,791)[37].

### Participant Characteristics and Enrollment

Enrollment will occur at primary (5) and referral (2) health care centers where the participant is seeking care for an illness which includes documented fever of 38°C or more at the time of enrollment as measured by temporal thermometry. Enrollment is to be restricted to individuals greater than ten years of age as it has previously been shown that respiratory tract infections cause > 70% of acute febrile illness in children ten years and under,[38] and will exclude illness of greater than 14 days of duration. Enrollment also excluded participants with clinical evidence of a focal cause of fever (such as symptoms of urinary tract infection, monoarticular septic arthritis, dental infection, wound or soft tissue infections, or a history of surgery in the past 30 days). Participation in recruitment areas where inpatient care occurs is restricted to the first 24 hours of admission. Health facility-based controls will be recruited within 14 days of the matched case. Those identified as controls will be enrolled within the health establishment where the study is being performed to attempt to match environmental and demographic risk exposure. Controls can be patients who do not have a measured or reported fever in the last ten days or have family members of cases in the AFI study. They will be age matched within 5 years of the age of the index case.

### Study Procedures

Enrolled participants will complete a questionnaire that collects information on the medical evaluation and clinical background of the patient, demographic, socioeconomic, occupational travel, and animal contact information. Additionally, biological samples, including whole blood, saliva, and mid-turbinate swabs, will be collected. A follow-up visit on days 21–28 after enrollment will be scheduled for both cases and controls in which participants provide additional clinical and household information as well as biological specimens (blood and saliva). Details of the procedures participants will undergo are shown in Table 1.

#### Laboratory Procedures

- *Biosafety*. Respiratory samples will be inactivated at 60°C for 30 minutes. Samples arriving at the laboratory, including saliva, blood, and mid-turbinate swabs, will be handled in a biosafety cabinet, and all laboratory personnel will wear personal protective equipment, including gloves, disposable aprons, and KN95 masks. Specimens not processed immediately will be stored at −80°C.
- *TaqMan Array Cards*. TaqMan array cards (Thermo Fisher Scientific, Waltham, MA) are a modular quantitative PCR diagnostic device that enables the detection of up to 32 pathogen targets in a single biological sample (Fig. 3). The card is essentially a microfluidic card that allows for the parallel processing of multiple (24) wells in which specific reagents for a single or multiplex assay are deposited in each individual well, and template and PCR common agents are loaded in a single step. Targets include: 1) the principal identified causes of acute febrile illness in the tropics; *Plasmodium falciparum, Plasmodium vivax,* dengue virus detection and serotyping assays, leptospirosis, tuberculosis, Salmonella typhi, *Streptococcus pneumonia,* and Brucella; 2) geographically relevant etiologies; *Trypanosoma cruzi,* Mayaro, Oropouche, Bartonella, *Orientia tustsugamushi,* Rickettsia, Hepatitis B and D; 3) diseases of importance in outbreaks; Yellow fever, Zika, Chikungunya, Equine Encephalitis virus, and 4) diseases of unknown importance in the region; Histoplasmosis, Hepatitis E, Yersinia pestis, Junin, Anaplasmosis, Borellia, Junin, Guaranito, Ehrlichiosis and West Nile Virus 5) chronic viral infections; HIV, CMV and EBV In this study, total nucleic acids will be extracted from whole blood samples using the High Pure Viral Nucleic Acid Large Volume Kit (Roche Life Science, Indianapolis, IN, USA) as instructed by manufacturers. TaqMan Fast Virus 1 - Step Master Mix (Thermo Fisher Scientific, Waltham, MA) will be used for the qPCR reactions performed in a QuantStudio 7 Flex. To increase the sensitivity of the assay, duplicate wells were run for all bacterial etiologies. Reactions with cycle thresholds (Ct) of less than or equal to 35 are considered positive, as supported by prior work to establish this cutoff as validated by sequencing [28, 30, 39]. Validation reactions include an internal control (MS2) to detect reaction inhibition and extraction blanks to detect contamination. The customized TAC utilized in this study has the potential to be redesigned and it is expected that individual targets may be added during the study period. Assay targets for the initial iteration are included in **Supplementary Table 1**.
  Results will be reviewed by two independent reviewers to ensure that interpretation is consistent. PCR targets with Ct’s of greater than 35 but less than 40 will be confirmed by single plex PCR and/or sequenced by Sanger sequencing as per Liu et al.[28].
- *SARS-CoV-2 and Influenza*. Mid-turbinate swabs from both cases and controls will be collected on Day 0 and on Day 21–28 and placed in 1.5 mL of T.E. buffer (pH 8.0). Mid-turbinate swabs obtained at Day 0 will be processed using the CDC Influenza SARS-CoV-2 (Flu SC2) multiplex assay that simultaneously detects SARS-CoV-2, influenza A, and Influenza B. Specifically, RNA will be extracted from 140 μL of the sample using the Qiaamp Viral RNA Mini Kit (Qiagen, Germantown, MD), as instructed by manufacturers. Primers and probes are detailed in **Supplementary Table 2.** The final assay consists of a 25 μL final reaction mixture with 6.25 μL of UltraPlex 1-Step ToughMix (Quantabio, Beverly, MA) (4X), forward and reverse primers (final reaction concentration of 1.6 μM, except for Influenza A Forward primers at 0.8 μM, Influenza A Reverse 1 primer at 1.2 μM and Influenza B Reverse 2 primer at 0.4 μM), probes (final reaction concentration of 0.4μM), 5.0 μL of RNA template and 7.75 μL of RNase and DNase free water (Ambion^™^, Thermo Fisher Scientific, Waltham, MA, USA). The assays will be performed on a QuantStudio 7 Flex (Applied Biosystems, Foster City, CA) using the following cycling conditions: 25°C for 2 minutes, 50°C for 15 minutes, 95°C for 3 minutes, followed by 45 cycles of 95°C for 15 seconds, and 60°C for 30 seconds. A negative control consisting of RNA and DNA-free water will be used for each extraction set. Positive controls are shown in **Supplementary Table 3**. Reactions with a Ct of less than 40 are considered positive Mid-turbinate swabs obtained at the follow-up visit will be processed only for SARS-CoV-2 using the CDC 2019-Novel Coronavirus (2019-nCoV) Real-Time RT-PCR Diagnostic Panel for specific assay steps; see Catalog # 2019-nCoVEUA-01. Positive controls are listed in **Supplementary Table 3.**

SARS-CoV-2 Variant Sequencing. All samples that are determined to be positive for SARS-CoV-2 will be sequenced to determine the variant in the population using the Illumina COVIDSeq Assay Kit per the manufacturer’s instructions. Briefly, each RNA sample will be converted to cDNA using 1st strand synthesis, and then the SARS-CoV-2 genome will be amplified with pre-designed primers that generate overlapping amplicon products of the genome. Next, Illumina adapters will be added to all PCR amplicons, and then all amplicons for a single sample will be pooled together and barcoded for multiplex sequencing. Next, all barcoded Illumina libraries will be pooled together, the libraries cleaned up and quantified using a Qubit 4 Fluorometer and normalized to 4 nM. Finally, the normalized Illumina sequence library was diluted to 75 pM and sequenced on an Illumina iSeq 100 using the v2 (300-cycle) reagent kit. Sequence reads were de-muliplexed according to the samples barcode, FASTQ files for the forward and reverse reads of each sample uploaded into Illumina’s Basespace (www.basespace.illumina.com), and the SARS-CoV-2 variant called using the DRAGEN COVID Lineage application (v3.5.7).

Dengue virus sequencing. All samples positive for dengue virus with Ct values less than 30 will be sequenced for targeted full-genome amplification and sequencing as per Cruz et al. Sequencing will be done using the MinION (Oxford Nanopore, Oxford, UK).

### Additional testing

Acute and convalescent sera allow for confirmatory testing using serological methods or targeted substudies to focus on pathogens where infectious load makes direct detection challenging *(Coxiella burnetti,* Rickettsiae spp).

### Statistical analysis and Power Calculation

**Figure 2:**
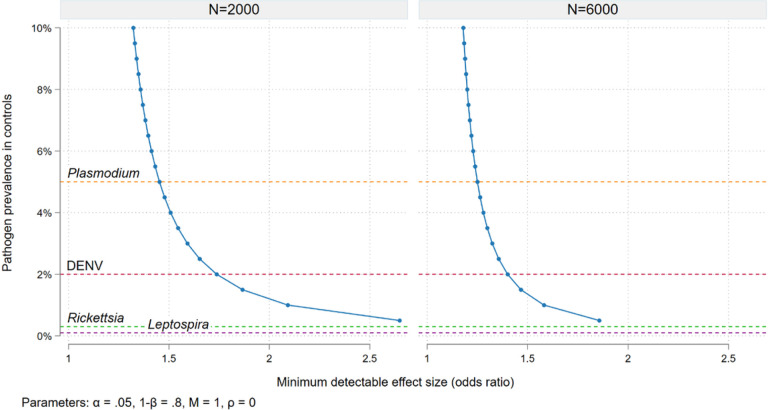
Power calculations - minimum effect size (odds ratio for acute febrile illness in cases compared to controls) detectable in the study overall (6,000 cases over 4 years) and for site-specific effects (2,000 cases over 4 years) given the prevalence of pathogen positivity in controls, 80% power, an alpha of 0.05 and 1:1 matching. Horizontal lines show documented prevalence of subclinical detection for 4 key pathogens.

**Figure 3:**
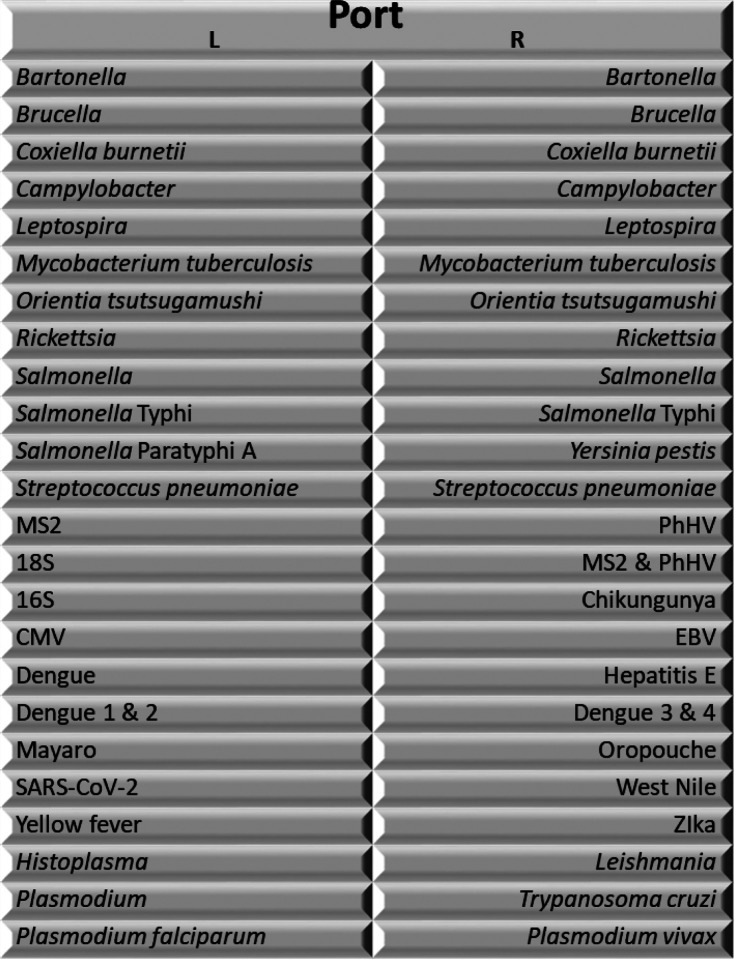
TaqMan Array Card

A primary aim of RIVERA is to quantify the burden of AFI attributable to each pathogen in a diagnostic panel, or the etiology-specific population attributable fraction (AFp). The inclusion of controls is necessary to estimate the associations between pathogen detection and AFI by etiology while adjusting for rates of subclinical infections. While crude, unadjusted AFps can be derived from standardly calculated odds ratios (ORs), maximum likelihood methods allow for adjustment for confounders and the presence of multiple pathogens, as well as the comparison of etiological and non-etiological (risk factor) exposures [40]. For analysis of RIVERA data, conditional logistic regression models will be fitted to the matched pairs data, treating AFI (case/control) status as the outcome and with pathogen-specific sample positivity as exposures to estimate the strength of association between pathogen detection and AFI while accounting for asymptomatic infections. Models will be further adjusted for sex, and other confounders, as well as other pathogens, to account for co-infections that are documented in this context (33,34). Adjusted attributable pathogen fractions (AFe) will be calculated from OR estimated by the models using the method proposed by Bruzzi and colleagues [41], which have since been adapted for applications to case-control data from studies of infectious syndromes of diverse etiology [42]. Assuming the absence of interactions between pathogens, the adjusted attributable fraction *AF*_*a*_ for a pathogen *A* is given by:

1 $$AF_{a}=\text{Pr}\left(A|AFI\right)\left(1-\frac{1}{OR_{a}}\right)$$

where *Pr(AIAFI)* represents the proportion of AFI cases in which *A* is present, and *OR*_*a*_ is the adjusted odds ratio for *A* estimated by the conditional logistic regression. Figure 2 graphs the relationship between the prevalence of a given pathogen in controls (the subclinical detection rate) and the minimum effect size detectable in the study overall (6,000 case/control pairs assuming 1,500 recruited per year for 4 years) and for the sub-group of site-specific estimates in 2000 pairs. With those numbers of subjects and considering, for example, a 5% *Plasmodium* prevalence in controls, we will be powered to detect a minimum odds ratio for AFI of approximately 1.25 in the study overall or 1.4 in sub-groups with 2,000 case/control pairs.

An alternative approach, proposed by Deloria Knoll and colleagues [43], will be used in parallel.

Despite the relative simplicity and ease of interpretability of the attributable fraction methods, the PERCH Integrated Analysis method, uses Bayesian nested partially latent class analysis to provide estimates at the individual- and population-level for each pathogen. These analytic methods are highly flexible and can be adapted to fit the intricacies of particular diagnostic test characteristics [43]. This approach has several advantages which include the ability to incorporate results from more than one test for a single pathogen into estimates (i.e. PCR and serology), for the adjustment of sensitivity and specificity of each test into the estimate, and for inclusion of pathogens in estimates which have population based odds ratio ≤ 1 [44].

In addition to etiological exposures (pathogens) we will also quantify population level attributable risk factors for exposure variables. Variables such as employment, history of travel by river, housing construction material, water storage and treatment, livestock husbandry, bush meat exposure, and domestic ectoparasite sightings. These will be tested for their unadjusted associations with AFI in univariable conditional logistic regression models and those for which the effect estimate is significant at the α < 0.05 level will be included alongside pathogen infection statuses in a final multivariable model, allowing both adjustments for confounding, and direct comparison of effect magnitudes between etiological and risk factor exposures to estimate the population attributable fractions for multiple risk factors. The association of clinical and laboratory features of the illness will also be examined to determine if these components can be associated with specific etiologies of AFI.

## Discussion

The results of this study will inform clinicians and public health practitioners as to the relative importance of a broad range of pathogens causing acute febrile illness in the Northern Amazon, a region of known importance in the identification and characterization of agents of novel and emerging infectious diseases. There are several commercial nucleic acid amplification assays designed for use in acute febrile illness etiology determination that aim to identify a broad set of etiologies. These include the BioFire FilmArray Global Fever Panel (19 pathogens)[45], Verigene, VerePLEX (26 pathogens)[46], and FeverDisk (12 pathogens) [47]. We selected custom-made cards as the ability to select pathogens of regional interest was seen as an important asset in responding to local clinicians’ needs assessment, and because acute febrile illness surveillance and emerging infectious diseases surveillance are overlapping tasks in our laboratory. We also selected a diagnostic method that would allow for the direct detection of the four major serotypes of dengue, as serotype switching is an important component of disease surveillance at the population level for epidemic response preparedness.

Nucleic acid-based methodologies yield certain clear advantages over older approaches that were based on aggregated results from culture, microscopy, lateral flow assays, and serology. First, the nucleic acid tests are in most cases more sensitive. Secondly, the streamlined approach in which most assays result from the testing of a single card facilitates reporting, permits on-site testing, and facilitates the clinical application of results. Lastly, nucleic acid isolated from the sample and from PCR amplification are each available for sequence-based confirmation and molecular epidemiology. This accelerates not only clinical care but reporting or results of notifiable diseases at the local, regional, national, and international level and is likely the only strategy that is truly feasible for emerging diseases surveillance and response in the modern era.

Independent of the diagnostic testing approach, the case control component will allow for the application of the attributable fraction approach for acute febrile illness for the primary analysis for disease burden estimates. In a planned secondary analysis, we will repeat estimates using a Bayesian approach. Advantages of this alternative approach allow for the integration of testing results from greater than one test per etiology, the integration of the sensitivity and specificity of the test into the estimate, and makes makes estimates at the individual rather than population level. As a result, etiologies that have fractions of <1 may appear in the etiologic distribution, as would be expected when predictions are individual rather than population level. Analysis of risk factors may also yield more efficient pathways for the improved clinical management of patients presenting with acute febrile illness in this region.

The team that has designed this study is composed of key stakeholders in the surveillance of infectious diseases in the region and clinicians overseeing the care of patients to enable the inclusion of appropriate populations maximally enable technical and de-identified data transfer at all levels of the project.

## Figures and Tables

**Table 1:** Study procedures performed on cases and controls

Participant Procedures	Day 0	Day 21–28
**Data**		
Medical Evaluation		
*a) Standardized case report format*	X	X
*b) Clinical signs*	X	X
*c) Vital signs*	X	X
*d) Laboratory findings*	X	
Questionnaires		
*Demographic and Socioeconomic information*	X	
*Occupational information*	X	
*Travel information*	X	
*Animal contact information*	X	
**Biological Specimens**		
Whole blood samples (18mL)	X	X
Saliva samples (3mL)	X	X
Mid-turbinate swab	X	X
**Diagnostic Tests**		
SARS-CoV-2 qPCR	X	X
Influenza A and B qPCR	X	
Multi-pathogen TAC	X	

## Data Availability

All other genomic data produced, including SARS-CoV-2 and dengue virus sequences, will be available under NCBI Bio project PRJNA813162. Additionally, SARS-CoV-2 sequences are also deposited in GISAID (10.55876/gis8.230315uv).

## Abbreviations

AFI: Acute Febrile Illness
AFe: Attributable pathogen fractions
CDC: Centers for Disease Control and Prevention, USA
Ct: Cycle threshold
DENV: Dengue virus
OR: Odds Ratios
PCR: Polymerase Chain Reaction
TAC: TaqMan Array Cards

## References

[1] Moreira J, Bressan CS, Brasil P, Siqueira AM: Epidemiology of acute febrile illness in Latin America. Clin Microbiol Infect 2018, 24(8):827–835. 29777926 10.1016/j.cmi.2018.05.001 PMC7172187

[2] Aramburu Guarda J, Ramal Asayag C, Witzig R: Malaria reemergence in the Peruvian Amazon region. Emerging infectious diseases 1999, 5(2):209–215. 10221872 10.3201/eid0502.990204 PMC2640690

[3] Anticona Huaynate CF, Pajuelo Travezano MJ, Correa M, Mayta Malpartida H, Oberhelman R, Murphy LL, Paz-Soldan VA: Diagnostics barriers and innovations in rural areas: insights from junior medical doctors on the frontlines of rural care in Peru. BMC Health Serv Res 2015, 15:454. 26438342 10.1186/s12913-015-1114-7 PMC4595324

[4] Hupalo DN, Luo Z, Melnikov A, Sutton PL, Rogov P, Escalante A, Vallejo AF, Herrera S, Arevalo-Herrera M, Fan Q : Population genomics studies identify signatures of global dispersal and drug resistance in Plasmodium vivax. Nature genetics 2016, 48(8):953–958. 27348298 10.1038/ng.3588 PMC5347536

[5] Parker BS, Paredes Olortegui M, Penataro Yori P, Escobedo K, Florin D, Rengifo Pinedo S, Cardenas Greffa R, Capcha Vega L, Rodriguez Ferrucci H, Pan WK et al: Hyperendemic malaria transmission in areas of occupation-related travel in the Peruvian Amazon. Malaria journal 2013, 12:178. 23724869 10.1186/1475-2875-12-178 PMC3673823

[6] Quispe AM, Llanos-Cuentas A, Rodriguez H, Clendenes M, Cabezas C, Leon LM, Chuquiyauri R, Moreno M, Kaslow DC, Grogl M et at: Accelerating to Zero: Strategies to Eliminate Malaria in the Peruvian Amazon. Am J Trop Med Hyg 2016, 94(6):1200–1207. 30851016 10.4269/ajtmh.15-0369 PMC4889734

[7] Reinbold-Wasson DD, Sardelis MR, Jones JW, Watts DM, Fernandez R, Carbajal F, Pecor JE, Calampa C, Klein TA, Turell MJ: Determinants of Anopheles seasonal distribution patterns across a forest to periurban gradient near Iquitos, Peru. Am J Trop Med Hyg 2012, 86(3):459–463. 22403317 10.4269/ajtmh.2012.11-0547 PMC3284362

[8] Witzig RS, Barker RH Jr.: Atypical Plasmodium vivax infection in Peru. Transactions of the Royal Society of Tropical Medicine and Hygiene 1994, 88(2):198. 8036671 10.1016/0035-9203(94)90293-3

[9] Stoddard ST, Wearing HJ, Reiner RC Jr., Morrison AC, Astete H, Vilcarromero S, Alvarez C, Ramal-Asayag C, Sihuincha, Rocha : Long-term and seasonal dynamics of dengue in Iquitos, Peru. PLoS neglected tropical diseases 2014, 8(7):e3003. 25033412 10.1371/journal.pntd.0003003 PMC4102451

[10] Liebman KA, Stoddard ST, Morrison AC, Rocha C, Minnick S, Sihuincha M, Russell KL, Olson JG, Blair PJ, Watts DM : Spatial dimensions of dengue virus transmission across interepidemic and epidemic periods in Iquitos, Peru (1999–2003). PLoS neglected tropical diseases 2012, 6(2):e1472. 22363822 10.1371/journal.pntd.0001472 PMC3283551

[11] Liebman KA, Stoddard ST, Reiner RC Jr., Perkins TA, Astete H, Sihuincha M, Halsey ES, Kochel TJ, Morrison AC, Scott TW: Determinants of heterogeneous blood feeding patterns by Aedes aegypti in Iquitos, Peru. PLoS neglected tropical diseases 2014, 8(2):e2702. 24551262 10.1371/journal.pntd.0002702 PMC3923725

[12] Kosek M, Lavarello R, Gilman RH, Delgado J, Maguina C, Verastegui M, Lescano AG, Mallqui V, Kosek JC, Recavarren S : Natural history of infection with Bartonella bacilliformis in a nonendemic population. The Journal of infectious diseases 2000, 182(3):865–872. 10950782 10.1086/315797

[13] Ganoza CA, Matthias MA, Saito M, Cespedes M, Gotuzzo E, Vinetz JM: Asymptomatic renal colonization of humans in the peruvian Amazon by Leptospira. PLoS neglected tropical diseases 2010, 4(2):e612. 20186328 10.1371/journal.pntd.0000612 PMC2826405

[14] Johnson MA, Smith H, Joeph P, Gilman RH, Bautista CT, Campos KJ, Cespedes M, Klatsky P Vidal C, Terry H : Environmental exposure and leptospirosis, Peru. Emerging infectious diseases 2004, 10(6):1016–1022. 15207052 10.3201/eid1006.030660 PMC3323149

[15] Matthias MA, Ricaldi JN, Cespedes M, Diaz MM, Galloway RL, Saito M, Steigerwalt AG, Patra KP Ore CV, Gotuzzo E : Human leptospirosis caused by a new, antigenically unique Leptospira associated with a Rattus species reservoir in the Peruvian Amazon. PLoS neglected tropical diseases 2008, 2(4):e213. 18382606 10.1371/journal.pntd.0000213 PMC2271056

[16] Russell KL, Montiel Gonzalez MA, Watts DM, Lagos-Figueroa RC, Chauca G, Ore M, Gonzalez JE, Moron C, Tesh RB, Vinetz JM: An outbreak of leptospirosis among Peruvian military recruits. Am J Trop Med Hyg 2003, 69(1):53–57. 12932097

[17] Kocher C, Jiang J, Morrison AC, Castillo R, Leguia M, Loyola S, Ampuero JS, Cespedes M, Halsey ES, Bausch DG : Serologic Evidence of Scrub Typhus in the Peruvian Amazon. Emerging infectious diseases 2017, 23(8):1389–1391. 28726619 10.3201/eid2308.170050 PMC5547797

[18] Kocher C, Morrison AC, Leguia M, Loyola S, Castillo RM, Galvez HA, Astete H, Flores-Mendoza C, Ampuero JS, Bausch DG : Rickettsial Disease in the Peruvian Amazon Basin. PLoS neglected tropical diseases 2016, 10(7):e0004843. 27416029 10.1371/journal.pntd.0004843 PMC4944934

[19] Loyola S, Flores-Mendoza C, Torre A, Kocher C, Melendrez M, Luce-Fedrow A, Maina AN, Richards AL, Leguia M: Rickettsia asembonensis Characterization by Multilocus Sequence Typing of Complete Genes, Peru. Emerging infectious diseases 2018, 24(5):931–933. 29664376 10.3201/eid2405.170323 PMC5938772

[20] Baisley KJ, Watts DM, Munstermann LE, Wilson ML: Epidemiology of endemic Oropouche virus transmission in upper Amazonian Peru. Am J Trop Med Hyg 1998, 59(5):710–716. 9840586 10.4269/ajtmh.1998.59.710

[21] Halsey ES, Siles C, Guevara C, Vilcarromero S, Jhonston EJ, Ramal C, Aguilar PV, Ampuero JS: Mayaro virus infection, Amazon Basin region, Peru, 2010–2013. Emerging infectious diseases 2013, 19(11):1839–1842. 24210165 10.3201/eid1911.130777 PMC3837653

[22] Santiago FW, Halsey ES, Siles C, Vilcarromero S, Guevara C, Silvas JA, Ramal C, Ampuero JS, Aguilar PV: Long-Term Arthralgia after Mayaro Virus Infection Correlates with Sustained Pro-inflammatory Cytokine Response. PLoS neglected tropical diseases 2015, 9(10):e0004104. 26496497 10.1371/journal.pntd.0004104 PMC4619727

[23] Bhaskaran D, Chadha SS, Sarin S, Sen R, Arafah S, Dittrich S: Diagnostic tools used in the evaluation of acute febrile illness in South India: a scoping review. BMC infectious diseases 2019, 19(1):970. 31722678 10.1186/s12879-019-4589-8 PMC6854686

[24] Chappuis F, Alirol E, d’Acremont V, Bottieau E, Yansouni CP: Rapid diagnostic tests for non-malarial febrile illness in the tropics. Clin Microbiol Infect 2013, 19(5):422–431. 23413992 10.1111/1469-0691.12154

[25] Blair PJ, Jiang J, Schoeler GB, Moron C, Anaya E, Cespedes M, Cruz C, Felices V, Guevara C, Mendoza L et at: Characterization of spotted fever group rickettsiae in flea and tick specimens from northern Peru. J Clin Microbiol 2004, 42(11):4961–4967. 15528680 10.1128/JCM.42.11.4961-4967.2004 PMC525230

[26] Blair PJ, Schoeler GB, Moron C, Anaya E, Caceda R, Cespedes M, Cruz C, Felices V, Guevara C, Huaman A : Evidence of rickettsial and leptospira infections in Andean northern Peru. Am J Trop Med Hyg 2004, 70(4):357–363. 15100447

[27] Forshey BM, Guevara C, Laguna-Torres VA, Cespedes M, Vargas J, Gianella A, Vallejo E, Madrid C, Aguayo N, Gotuzzo E : Arboviral etiologies of acute febrile illnesses in Western South America, 2000–2007. PLoS neglected tropical diseases 2010, 4(8):e787. 20706628 10.1371/journal.pntd.0000787 PMC2919378

[28] Liu J, Ochieng C, Wiersma S, Stroher U, Towner JS, Whitmer S, Nichol ST, Moore CC, Kersh GJ, Kato C : Development of a TaqMan Array Card for Acute-Febrile-Illness Outbreak Investigation and Surveillance of Emerging Pathogens, Including Ebola Virus. J Clin Microbiol 2016, 54(1):49–58. 26491176 10.1128/JCM.02257-15 PMC4702733

[29] Liu J, Kabir F, Manneh J, Lertsethtakarn P Begum S, Gratz J, Becker SM, Operario DJ, Taniuchi M, Janaki L : Development and assessment of molecular diagnostic tests for 15 enteropathogens causing childhood diarrhoea: a multicentre study. Lancet Infect Dis 2014, 14(8):716–724. 25022434 10.1016/S1473-3099(14)70808-4

[30] Marks F, Liu J, Soura AB, Gasmelseed N, Operario DJ, Grundy B, Wieser J, Gratz J, Meyer CG, Im J : Pathogens That Cause Acute Febrile Illness Among Children and Adolescents in Burkina Faso, Madagascar, and Sudan. Clin Infect Dis 2021, 73(8):1338–1345. 33822011 10.1093/cid/ciab289 PMC8528393

[31] Smith T, Genton B, Baea K, Gibson N, Taime J, Narara A, Al-Yaman F, Beck HP Hii J, Alpers M: Relationships between Plasmodium falciparum infection and morbidity in a highly endemic area. Parasitology 1994, 109 (Pt 5):539–549. 7831089 10.1017/s0031182000076411

[32] Smith T, Schellenberg JA, Hayes R: Attributable fraction estimates and case definitions for malaria in endemic areas. Stat Med 1994, 13(22):2345–2358. 7855468 10.1002/sim.4780132206

[33] Ly S, Fortas C, Duong V, Benmarhnia T, Sakuntabhai A, Paul R, Huy R, Sorn S, Nguon K, Chan S : Asymptomatic Dengue Virus Infections, Cambodia, 2012–2013. Emerging infectious diseases 2019, 25(7):1354–1362. 31211672 10.3201/eid2507.181794 PMC6590774

[34] Haby MM, Pinart M, Elias V, Reveiz L: Prevalence of asymptomatic Zika virus infection: a systematic review. Bull World Health Organ 2018, 96(6):402–413D. 29904223 10.2471/BLT.17.201541 PMC5996208

[35] Shih DC, Silver R, Henao OL, Alemu A, Audi A, Bigogo G, Colston JM, Edu-Quansah EP, Erickson TA, Gashu A : Incorporating COVID-19 into Acute Febrile Illness Surveillance Systems, Belize, Kenya, Ethiopia, Peru, and Liberia, 2020–2021. Emerging infectious diseases 2022, 28(13):S34–S41. 10.3201/eid2813.220898 PMC9745219 36502419

[36] Peru INdEyId: Comportamiento de la Mortalidad Infantil por departamento, vol. 4; 2017.

[37] Peru INdEyId: INEI: Health thematic index. In.; 2022.

[38] D’Acremont V, Kilowoko M, Kyungu E, Philipina S, Sangu W, Kahama-Maro J, Lengeler C, Cherpillod P Kaiser L, Genton B: Beyond malaria-causes of fever in outpatient Tanzanian children. N Engl J Med 2014, 370(9):809–817. 24571753 10.1056/NEJMoa1214482

[39] Moore CC, Jacob ST, Banura P Zhang J, Stroup S, Boulware DR, Scheld WM, Houpt ER, Liu J: Etiology of Sepsis in Uganda Using a Quantitative Polymerase Chain Reaction-based TaqMan Array Card. Clin Infect Dis 2019, 68(2):266–272. 29868873 10.1093/cid/ciy472 PMC6321855

[40] Sjolander A, Vansteelandt S: Doubly robust estimation of attributable fractions. Biostatistics 2011, 12(1):112–121. 20719781 10.1093/biostatistics/kxq049

[41] Bruzzi P Green SB, Byar DP Brinton LA, Schairer C: Estimating the population attributable risk for multiple risk factors using case-control data. Am J Epidemiol 1985, 122(5):904–914. 4050778 10.1093/oxfordjournals.aje.a114174

[42] Blackwelder WC, Biswas K, Wu Y, Kotloff KL, Farag TH, Nasrin D, Graubard BI, Sommerfelt H, Levine MM: Statistical methods in the Global Enteric Multicenter Study (GEMS). Clin Infect Dis 2012, 55 Suppl 4:S246–253. 23169937 10.1093/cid/cis788 PMC3502316

[43] Deloria Knoll M, Fu W, Shi Q, Prosperi C, Wu Z, Hammitt LL, Feikin DR, Baggett HC, Howie SRC, Scott JAG : Bayesian Estimation of Pneumonia Etiology: Epidemiologic Considerations and Applications to the Pneumonia Etiology Research for Child Health Study. Clin Infect Dis 2017, 64(suppl_3):S213–S227. 28575370 10.1093/cid/cix144 PMC5447849

[44] Hammitt LL, Feikin DR, Scott JAG, Zeger SL, Murdoch DR, O’Brien KL, Deloria Knoll M: Addressing the Analytic Challenges of Cross-Sectional Pediatric Pneumonia Etiology Data. Clin Infect Dis 2017, 64(suppl_3):S197–S204. 28575372 10.1093/cid/cix147 PMC5447845

[45] Manabe YC, Betz J, Jackson O, Asoala V, Bazan I, Blair PW, Chang A, Chusri S, Crump JA, Edgel KA : Clinical evaluation of the BioFire Global Fever Panel for the identification of malaria, leptospirosis, chikungunya, and dengue from whole blood: a prospective, multicentre, cross-sectional diagnostic accuracy study. Lancet Infect Dis 2022, 22(9):1356–1364. 35716700 10.1016/S1473-3099(22)00290-0 PMC9420791

[46] Tan JJ, Capozzoli M, Sato M, Watthanaworawit W, Ling CL, Mauduit M, Malleret B, Gruner AC, Tan R, Nosten FH : An integrated lab-on-chip for rapid identification and simultaneous differentiation of tropical pathogens. PLoS neglected tropical diseases 2014, 8(7):e3043. 25078474 10.1371/journal.pntd.0003043 PMC4117454

[47] Hin S, Lopez-Jimena B, Bakheit M, Klein V, Stack S, Fall C, Sall A, Enan K, Mustafa M, Gillies L : Fully automated point-of-care differential diagnosis of acute febrile illness. PLoS neglected tropical diseases 2021, 15(2):e0009177. 33630852 10.1371/journal.pntd.0009177 PMC7906357

